# Evaluation of Early and Late Effects into the Acute Spinal Cord Injury of an Injectable Functionalized Self-Assembling Scaffold

**DOI:** 10.1371/journal.pone.0019782

**Published:** 2011-05-18

**Authors:** Daniela Cigognini, Alessandro Satta, Bianca Colleoni, Diego Silva, Matteo Donegà, Stefania Antonini, Fabrizio Gelain

**Affiliations:** 1 Biotechnology and Biosciences Department, University of Milano-Bicocca, Milano, Italy; 2 Center for Nanomedicine and Tissue Engineering, Niguarda Ca' Granda Hospital, Milano, Italy; 3 Casa Sollievo della Sofferenza Hospital, San Giovanni Rotondo, Italy; Massachusetts Institute of Technology, United States of America

## Abstract

The complex physiopathological events occurring after spinal cord injury (SCI) make this devastating trauma still incurable. Self-assembling peptides (SAPs) are nanomaterials displaying some appealing properties for application in regenerative medicine because they mimic the structure of the extra-cellular matrix (ECM), are reabsorbable, allow biofunctionalizations and can be injected directly into the lesion. In this study we evaluated the putative neurorigenerative properties of RADA16-4G-BMHP1 SAP, proved to enhance *in vitro* neural stem cells survival and differentiation. This SAP (RADA16-I) has been functionalized with a bone marrow homing motif (BMHP1) and optimized via the insertion of a 4-glycine-spacer that ameliorates scaffold stability and exposure of the biomotifs. We injected the scaffold immediately after contusion in the rat spinal cord, then we evaluated the early effects by semi-quantitative RT-PCR and the late effects by histological analysis. Locomotor recovery over 8 weeks was assessed using Basso, Beattie, Bresnahan (BBB) test. Gene expression analysis showed that at 7 days after lesion the functionalized SAP induced a general upregulation of GAP-43, trophic factors and ECM remodelling proteins, whereas 3 days after SCI no remarkable changes were observed. Hystological analysis revealed that 8 weeks after SCI our scaffold increased cellular infiltration, basement membrane deposition and axon regeneration/sprouting within the cyst. Moreover the functionalized SAP showed to be compatible with the surrounding nervous tissue and to at least partially fill the cavities. Finally SAP injection resulted in a statistically significant improvement of both hindlimbs' motor performance and forelimbs-hindlimbs coordination. Altogether, these results indicate that RADA16-4G-BMHP1 induced favourable reparative processes, such as matrix remodelling, and provided a physical and trophic support to nervous tissue ingrowth. Thus this biomaterial, eventually combined with cells and growth factors, may constitute a promising biomimetic scaffold for regenerative applications in the injured central nervous system.

## Introduction

Regenerative medicine features the valuable potential to cure a broad range of diseases and injuries, including the degeneration of central nervous system (CNS), in which the progressive death of nervous cells results in permanent disabilities and/or cognitive deficits. Spinal cord injuries (SCI) are among the most devastating pathologies of CNS, leading to partial or complete loss of respiratory, autonomic and sensory-motor functions [1]. In spite of the growing knowledge of the processes involved in SCI, the treatments available for these patients are still unable to restore the lost functions and are mainly limited to the administration of anti-inflammatory agents (i.e. methylprednisolone), surgical decompression of spinal cord, stabilization of vertebral column, rehabilitation and functional electrical stimulation (FES) [2], [3], [4]. The poor outcome of therapies aiming to cure SCI is due to the complexity of the physiopathological events that occur after this trauma. These unfavourable events, including invasion of inflammatory and glial cells, secretion of inhibitors of axon growth, ongoing apoptosis of neural cells and demyelination and formation of cavities or cysts, culminate in glial scarring and loss of the complex nervous cytoarchitecture [5], [6], [7]. To contrast these processes and favour neuroprotection and/or nervous tissue regeneration, over the past decades various strategies have been proposed, including cell transplantation, scaffold implantation, drug delivery and rehabilitative training [5], [8], [9].

Up to now, cellular transplantation alone led to moderate sensory and motor improvements in animal models of SCI and failed in reconstituting a functional tissue in large lesions because the area of cyst and glial scarring constitutes a gap and a physical barrier to regeneration [10], [11]. In order to bridge the tissue defect, various biomaterials, either naturally derived or synthetic, have been tested [12]. Hydrogels are biomaterials characterized by a porous network of polymeric nano- and microfibers featuring three-dimensional (3D) microenvironments *in vitro* and *in vivo* [13]. Common naturally derived hydrogel materials used in stem cell-based research and in experimental models of SCI include collagen [14], hyaluronic acid [15], methylcellulose [16] and agarose [17], eventually functionalized with a laminin motif [18]. Even if these biomaterials showed to support axon regeneration and myelination and to reduce cavity formation, synthetic polymers permit to more easily and precisely adjust key parameters of the scaffold (3D architecture, porosity, stiffness, degradation rate, etc) and to add functionalizations [13]. Scaffold modifications with functional motifs can be useful for enhancing cell adhesion [19], achieving slow release of molecules and/or cells [20], [21], controlling the spatiotemporal differentiation of transplanted stem cells [22], [23], [24] and for ameliorating the biocompatibility of the implant [25], [26].

Among the most used synthetic hydrogels there are self-assembling peptides (SAPs). They are composed of short, repeating units of amino acids that form nanofibrous scaffolds in response to thermal or pH changes [27]. The ability to form a rigid scaffold only at physiological pH or specific salt concentrations makes SAPs appealing for application in SCI because they can be injected directly into the lesion site, then minimizing the damage brought to the cord by scaffold implantation surgery. At microscopic level SAPs mimic the porosity and the nanostructure of the extra-cellular matrix (ECM), thus permitting the cells to reside in a 3D environment; moreover, they allow biofunctionalizations suited for the desired application [28]. Thanks to these properties, SAPs-based scaffolds have been successfully used for a large variety of applications, including 3D cell cultures [29], tissue engineering [30] and regenerative medicine applications [31]. The most commonly employed SAP is RADA16-I [27], [31], [32].

We previously demonstrated that RADA16-I, functionalized with the sequence PFSSTKT, significantly enhanced *in vitro* survival and differentiation of adult mouse neural stem cells (NSC) [33].

PFSSTKT (BMHP1) belongs to a class of bone marrow homing peptides (BMHP) that were identified by applying the phage display methodology to the bone marrow and stem cells: Becker and colleagues found a family of heptapeptides -mainly consisting of lysine, proline, phenylalanine, two serines and two threonines- which specifically homed to bone marrow and bound to primitive hematopoietic stem cells [34]. Even if function of the sequence PFSSTKT still remains to be elucidated, its observed biological effect on NSCs is probably due to the sharing of some differentiating pathways and adhesion receptors between bone marrow stem cells and NSCs [35].

Recently we showed that RADA16-I functionalized with BMHP1 fostered conspicuous nervous tissue regrowth in chronic SCI in rats [36]. In this study we injected the SAP, loaded with cytokines, into electrospun nanofiber channels, then showing that by engineering SAP-based matrices into neuroprosthetics it is possible to replace large hollow tissue gaps in the chronically injured spinal cord.

However, despite these promising results, later on, we demonstrated that the insertion of four glycines between the self-assembling core of RADA16-I and the functional motif BMHP1 increased the nanostructure stability of SAPs and the exposure of the functional motif, improving the *in vitro* adhesion, viability and differentiation of mouse NSCs in comparison with functionalized SAP featuring shorter spacers [37].

In order to evaluate the putative application for neuroregenerative purposes of this latter SAP, named RADA16-4G-BMHP1, we delivered it in the lesion site of acutely contused rat spinal cords and we evaluated its early and late effects on injured tissues and motor function recovery. The obtained data showed that the injection of RADA16-4G-BMHP1 immediately after injury did not prevent cyst formation but enhanced nervous tissue ingrowth. RT-PCR assay displayed that the scaffold induced a favourable matrix remodelling process and provided a physical and trophic support to tissue regrowth, then resulting in an increased cellular infiltration and axon regeneration/sprouting, as seen by histological analysis at 8 weeks after SCI. Moreover the scaffold proved to be compatible with the nervous tissue and to ameliorate the locomotor recovery of animals.

## Results

We randomly divided 45 female Sprague-Dawley rats into 3 groups as follows: 1) injured animals (SCI control group); 2) animals receiving contusion and injection of saline solution (saline control group); 3) animals receiving contusion and injection of the functionalized SAP (4G-BMHP1 treatment group). RADA16-I-4G-BMHP1 self-assembling peptide solution (1% w/v) was sonicated for 30 min prior usage (see methods for details).

### Gene expression analysis of early effects induced by the scaffold after SCI

**Figure 1:**
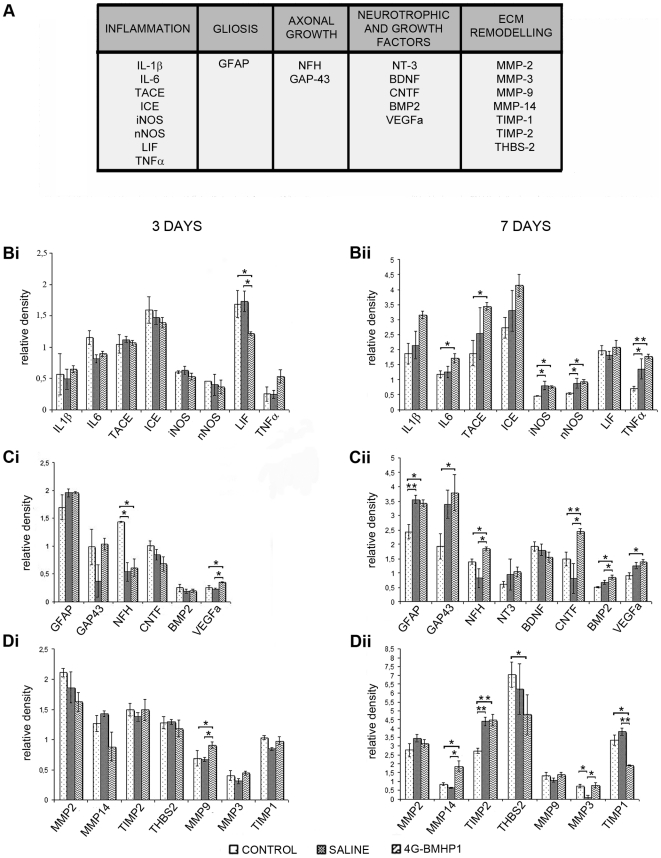
Gene expression analysis in the acute and subacute phases of spinal cord injury. Semi-quantitative RT-PCR was employed to evaluate mRNA expression of several genes involved in destructive and reparative processes following SCI (A). The relative expression of genes was determined by measuring the band intensity and using cyclophilin as housekeeping (relative density). Overall, at 3 days after SCI few differences were observed among treatment (4G-BMHP1) and both control (saline and SCI control) groups (Bi, Ci, Di); NT3, BDNF and NGF were undetectable in all groups. At 7 days after SCI there was a general upregulation of inflammatory genes in both injected groups (biomaterial or saline) in comparison with SCI control group (Bii), while a general mRNA overexpression for GAP-43, neurotrophins, growth factors (Cii) and ECM remodelling proteins (Dii) was observed only in the treatment group in comparison with one or both control groups; NGF was undetectable in all groups. Values represent means ± SEM. Significance symbols: * p<0.05, ** p<0.001.

Early effects induced by the injection of RADA16-I-4G-BMHP1 at the injury site were assessed by a semi-quantitative RT-PCR assay. Changes in the mRNA expression of genes involved in the inflammatory process, secretion of neurotrophic factors, matrix remodelling, gliosis and nervous tissue repair (Figure 1A) were evaluated at 3 and 7 days post injury (dpi). Gene names and characteristics of gene-specific primers are reported in Table 1.

**Table 1:** Characteristics of gene-specific primers for semi-quantitative RT-PCR.

Gene name	Accession n.^1^	Primer Sequence 5′–3′	Ampliconlength (bp)	Cycle^2^
cyclophilin A	XM_345810	F: gacaaagttccaaagacagca	470	22
		R: ctgagctacagaaggaatggtttga		
interleukin 1 beta (IL1β)	NM_031512	F: tcgggaggagacgactctaa	201	33
		R: gaaagctgcggatgtgaagt		
interleukin 6 (IL6)	NM_012589	F: ccggagaggagacttcacag	161	34
		R: acagtgcatcatcgctgttc		
tumor necrosis factor alpha (TNFα)	NM_012675	F: tactgaacttcggggtgatcggtcc	557	35
		R: cagagcaatgactccaaagta		
caspase 1 (ICE)	NM_012762	F: cacattgaagtgcccaagct	245	32
		R: tccaagtcacaagaccaggc		
ADAM metallopeptidase domain 17 (TACE)	NM_020306	F: gttacaactcatgaattggg	372	33
		R: gacagtttttacagcaagg		
neuronal nitric oxide synthase 1 (nNOS)	NM_052799	F: gaataccagcctgatccatgcaa	602	35
		R: tcctccaggagggtgtccaccgcatg		
inducible nitric oxide synthase 2 (iNOS)	NM_012611	F: acctgggaacacctggggatttt	565	32
		R: tgggtcttcgggcttcaggtt att		
bone morphogenetic protein 2 (BMP2)	NM_017178	F: acacagggacacaccaaccat	125	29
		R: tgtgaccagctgtgttcatcttg		
ciliary neurotrophic factor (CNTF)	NM_013166	F: aacacctctgacccttcacc	193	29
		R: tgcctcagtcatctcactcc		
brain-derived neurotrophic factor (BDNF)	NM_012513	F: tcagtggctggctctcatac	214	34
		R: aggacggaaacagaacgaac		
leukemia inhibitory factor (LIF)	NM_022196	F: aacgtggaaaagctatgtgcg	101	33
		R: gcgaccatccgatacagctc		
nerve growth factor (NGF)	XM_227525	F: gcccactggactaaacttcagc	349	34
		R: ccgtggctgtggtcttatctc		
neurotrophin 3 (NT-3)	NM_031073	F: tgcagagcataagagtcacc	269	35
		R: aagtcagtgctcggacgtag		
vascular endothelial growth factor A (VEGFa)	NM_031836	F: ccatgaactttctgctctctt	482	35
		R: ggtgagaggtctagttccc		
matrix metallopeptidase 9 (MMP9)	NM_031055	F: cccaaagacctgaaaacctcc	98	33
		R: ttctctcccatcatctgggc		
matrix metallopeptidase 3 (MMP3)	NM_133523	F: ttgtccttcgatgcagtcag	126	35
		R: agacggccaaaatgaagaga		
TIMP metallopeptidase inhibitor 1 (TIMP1)	NM_053819	F: gtgcacagtgtttccctgtt	103	31
		R: ctggtagcccttctcagagc		
matrix metallopeptidase 2 (MMP2)	NM_031054	F: tacgatgatgaccggaagtgg	101	35
		R: gagtgttccagccccatgg		
matrix metallopeptidase 14 (MMP14)	NM-031056	F: cactgctggacaaggtctg	413	35
		R: cctgaggtcatagttcagag		
TIMP metallopeptidase inhibitor 2 (TIMP2)	NM_021989	F: gcatcacccagaagaagagc	174	32
		R: tgatgcaggcaaagaacttg		
thrombospondin 2 (THBS2)	NM_001169138	F: ggatgtacgtggccaaggg	641	35
		R: ctgggtcccagagccaca		
glial fibrillary acidic protein (GFAP)	NM_017009	F: tacagacaggaggcggatgaagcc	418	27
		R: gcatttgcctctccaaggactc		
growth associated protein 43 (GAP-43)	NM_008083	F: gggagatggctctgctact	482	35
		R: agacagggttcaggtggg		
neurofilament, heavy polypeptide (NFH)	NM_012607	F:atcgctgcttacagaaaactcc	182	29
		R: tcctctacaatgacggtttcct		

1 NCBI accession number of mRNA and corresponding gene, available at http://www.ncbi.nlm.nih.gov/gene.

2 cycle number corresponding to the exponential phase of amplification of the PCR product using 50 ng of cDNA.

Several genes involved in the inflammatory response were analyzed in order to evaluate if the scaffold could attenuate or enhance the inflammatory events following SCI. These genes codify for the pro-inflammatory cytokines IL-1β, IL-6, LIF and TNFα, for TACE and ICE, that are two enzymes involved in the proteolytic activation of TNFα and IL-1β respectively, and for iNOS and nNOS, which are two enzymes responsible for the production of nitric oxide. At 3 dpi there were no significant differences in mRNA expression of these genes among groups, with the exception of LIF, that had lower mRNA levels in the 4G-BMHP1 group in comparison to both control groups (Figure 1Bi). At 7 dpi, mRNA levels of LIF were similar in all groups, while a significant increase of the mRNA expression for IL-6, TNFα, TACE, nNOS and iNOS appeared in the treatment group in comparison with SCI control group (Figure 1Bii). As the upregulation of TNFα, iNOS and nNOS was also observed in the saline control group, we can suppose that this slight increment of the sub-acute inflammatory response could be due to the injection procedure. The presence of the biomaterial seemed to induce a further little increase of the inflammatory response in comparison with saline-injected group, as showed by the upregulation of IL-6 and TACE only in the treatment group.

The second group of genes we studied consisted of the neuronal cytoskeletal protein NFH- the heavy subunit of neurofilaments (NF)- and GAP-43, which is a phosphoprotein associated with axonal growth cones and expressed at high levels by neurons during axon growth [38]; moreover we assessed the mRNA expression of GFAP, a protein of the cytoskeleton of astrocytes. As NFH mRNA expression declined at 3 days in both injected groups (4G-BMHP1 and saline) in comparison with SCI control group (Figure 1Ci), we can suppose that the needle insertion could have caused a slight damage of nervous tissue and exacerbated the NF expression decrease typically seen after SCI [39]. However, at 7 dpi the 4G-BMHP1 group showed higher mRNA levels than saline-injected and SCI control groups (Figure 1Cii), then suggesting that already 1 week after injection the scaffold could have played a role in the regeneration of the injured nervous tissue [40]. This hypothesis seems to be supported by the concurrent upregulation of GAP-43 mRNA expression that was observed only in the treatment group (Figure 1Cii). GFAP mRNA levels were higher in both groups subjected to injection in comparison with SCI control group, indicating that at 7 dpi this procedure may induce a bigger gliosis.

Third group of genes we considered included the neurotrophic factors NGF, NT3, BDNF and CTNF and the growth factors BMP2 and VEGF. CNTF is involved in CNS neural repair and its levels have been showed to increase after SCI [41]; BMP2 promotes astrogliogenesis during CNS development and its expression is upregulated after SCI, then participating to glial scar formation [42]; VEGF is a potent stimulator of angiogenesis, moreover it seems to provide a neuroprotective effect on neurons after injury [43]. At 3 dpi mRNA expression of these genes was similar -or undetectable- in all groups, with the exception of VEGF, which was upregulated in 4G-BMHP1 group in comparison with both control groups (Figure 1Ci). At 7 dpi a higher mRNA expression of CNTF, BMP2 and VEGF was observed in the treatment group in comparison with SCI control group and, for CNTF and BMP2, in comparison with saline control group too (Figure 1Cii). These data suggest that the scaffold could stimulate endogenous repair processes via VEGF and CNTF.

Finally we analysed several genes involved in matrix remodelling. MMP-9 and MMP-2 are metalloproteinases (MMPs) involved in the neuroinflammation and remodelling of the neural ECM. After SCI, MMPs are up-regulated and initially they are involved in the disruption of the blood-spinal cord barrier [44], successively they participate in regenerative processes like angiogenesis and axonal sprouting/regrowth [45], [46]. MMP-3 and MMP-14 are the proteolytic activators of MMP-9 and MMP-2 respectively, whereas TIMP-1 and TIMP-2 are two inhibitor proteins: they bind MMP-9 and MMP-2 respectively and block their cleavage [47]. THBS-2 is another inhibitor of MMP-2 [47]. At 3 dpi MMP-9 mRNA was more expressed in the 4G-BMHP1 group in comparison with both controls (Figure 1Di). At 7 dpi the metalloproteinases inhibitors THBS-2 and TIMP-1 were found downregulated in the treatment group in comparison with SCI control group or both controls, respectively; in line with these data, in the treatment group the mRNA levels of the metalloproteinases activators MMP-14 and MMP-3 were higher than both controls or only saline group, respectively (Figure 1Dii). Overall these results suggest that the scaffold promoted the metalloproteinases activity. It remains to clarify the role of TIMP-2, that was found upregulated in 4G-BMHP1- and saline-injected groups in comparison with SCI control group: even if TIMP-2 inhibits a broad range of MMPs, some authors have suggested that it may have a role also as activator of MMP-2 [47].

### Hystological analysis of reparative processes induced by the scaffold

The late effects induced by the injection of RADA16-I-4G-BMHP1 were assessed by histological analysis of the injury site. Eight weeks after SCI we quantified the cyst and cavities dimension, axon sprouting/regeneration and macrophage infiltration, moreover we evaluated the vascularisation, gliosis and axon maturation (see methods for details).

**Figure 2:**
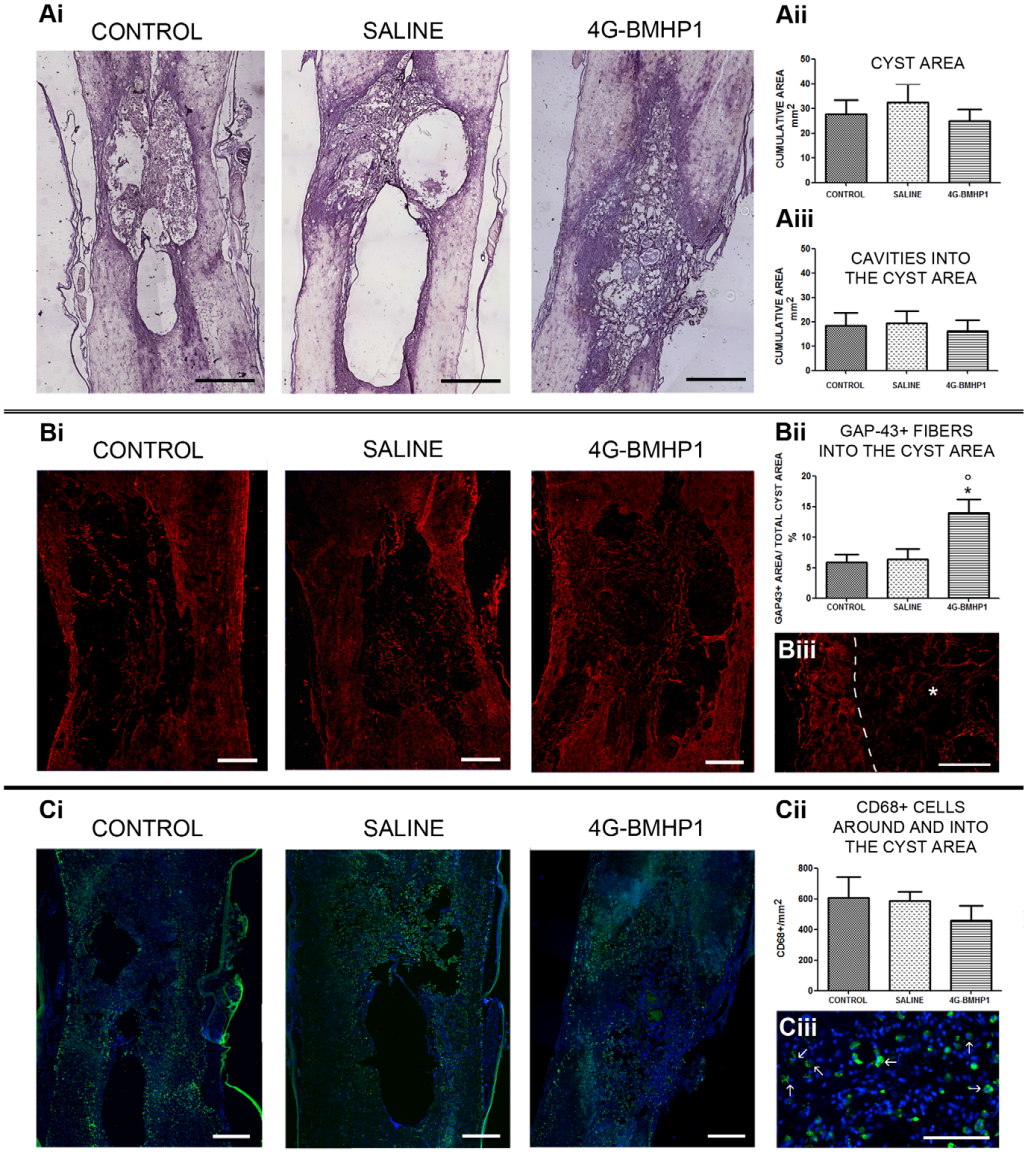
Quantitative histological analysis in the chronic phase of SCI. (A): lesion size was quantified on spinal cord longitudinal sections stained with hematoxylin/eosin (Ai) and it was reported as cumulative area (mm^2^). No significant differences among groups were found when measuring both the whole cyst area (Aii) and the cavities into the cyst area (Aiii). Scale bar  = 800 µm. (B): GAP-43 positive fibers (Bi and Biii, red) were measured on six longitudinal sections after immunofluorescence staining and the values were expressed as percentage of the total area of the cyst. The GAP-43 immunopositive area was significantly higher in biomaterial-treated group (4G-BMHP1) than both control groups (saline and SCI control) (Bii). In Biii the positive GAP-43 signal is showed at higher magnification (asterisk and dotted line indicate the cyst and its border, respectively). Scale bar  = 400 µm in Bi, 50 µm in Biii. (C): CD68 positive cells (Ci and Ciii, green) were counted on three longitudinal sections after immunofluorescence staining and reported as cumulative number per mm^2^. Nuclei were counterstained with DAPI. Macrophage infiltration was observed in the tissue surrounding the cyst and into the cavities of all groups (Cii). In Ciii, at higher magnification, we reported an image representative of the CD68 positive cells (arrows) we observed in all groups. Scale bar  = 400 µm in Bi, 100 µm in Biii. Values represent means ± SEM. Significance symbols: * p<0.05, 4G-BMHP1 vs SCI control; ° p<0.05, 4G-BMHP1 vs saline.

Cyst and cavities extent was measured on hematoxylin-eosin stained sections (Figure 2Ai). Average area of the whole cyst was similar in all groups (Figure 2Aii); likewise, no significant differences were observed among treatment and both control groups when we measured only the cavities size within the cyst excluding strands of connective tissue (*trabeculae*) (Figure 2Aiii).

In order to quantify axon regeneration/sprouting across the cyst we investigated the presence of GAP-43 positive fibers and we expressed the relative value of GAP-43 immunopositive area as percentage of the total cyst area (Figure 2Bi). Interestingly, we found a significantly greater synthesis of GAP-43 in 4G-BMHP1 group (12.94±2.03% of the whole cyst area) in comparison with saline (6.33±1.7% of the whole cyst area) and SCI control (5.84±1.29% of the whole cyst area) groups (Figure 2Bii).

The chronic inflammatory response was evaluated by counting macrophages into the lesion site (Figure 2Ci). We observed several infiltrating CD68 immunopositive cells in all groups, indicating that at this late phase of the injury the scaffold didn't significantly affect the host immune response (Figure 2Cii).

**Figure 3:**
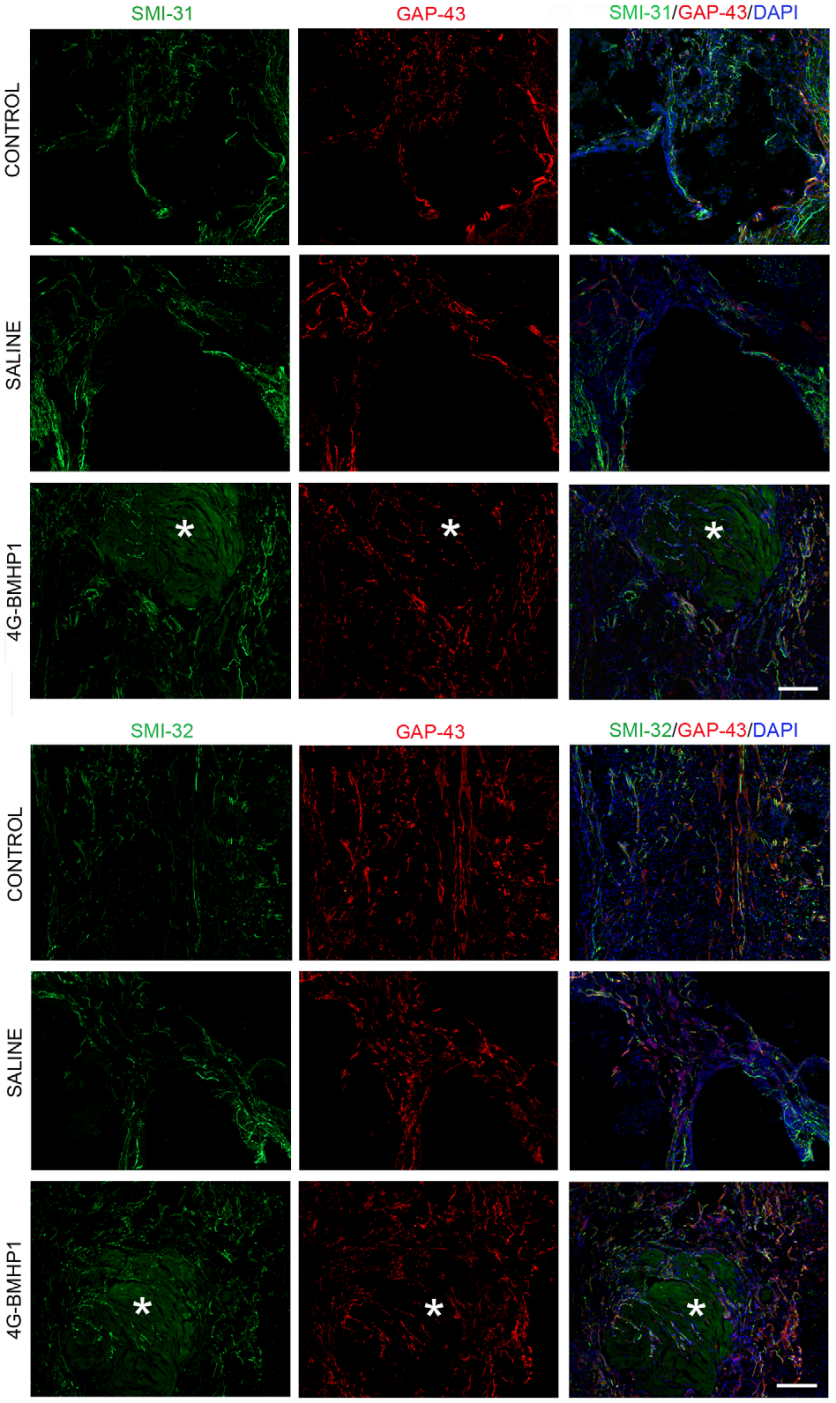
Evaluation of mature and immature nerve fibers infiltrating the cyst. Images show the cyst cavity in SCI control, saline injected and 4G-BMHP1 treated animals at 8 weeks after lesion. Asterisks indicate the scaffold. Immunolabelling for GAP-43 (red) coupled with SMI-31 (green) or SMI-32 (green) was made on longitudinal sections. Nuclei were counterstained with DAPI. Within the cyst, the majority of GAP-43^+^ sprouting/growing axons consisted of immature fibers expressing the non-phosphorylated neurofilament H (SMI-32^+^), even if also GAP-43^+^ mature fibers, staining for the phosphorylated neurofilament H (SMI-31^+^), were observed. The percentage of GAP-43^+^ fibers expressing either SMI-32 or SMI-32 appeared similar in all groups. Scale bar  = 200 µm.

The nature of neural fibers infiltrating the cyst was further investigated in all experimental groups by performing the immunofluorescent labelling with β-TubIII, a neuronal marker, MBP, which detects myelin, and SMI-31 and SMI-32, that react respectively with phosphorylated and nonphosphorylated neurofilament H of axons. Within the cyst, the majority of β-TubIII positive fibers also expressed GAP-43, whereas some of them were associated with MBP, indicating that myelination occurred at 8 weeks post-treatment. Among GAP-43 positive fibers, the majority of them expressed SMI-32, even if another part of GAP-43 positive fibers stained with SMI-31, showing that among growing axons there were both immature (SMI-32^+^) [48] and mature (SMI-31^+^) [49] fibers (Figure 3).

**Figure 4:**
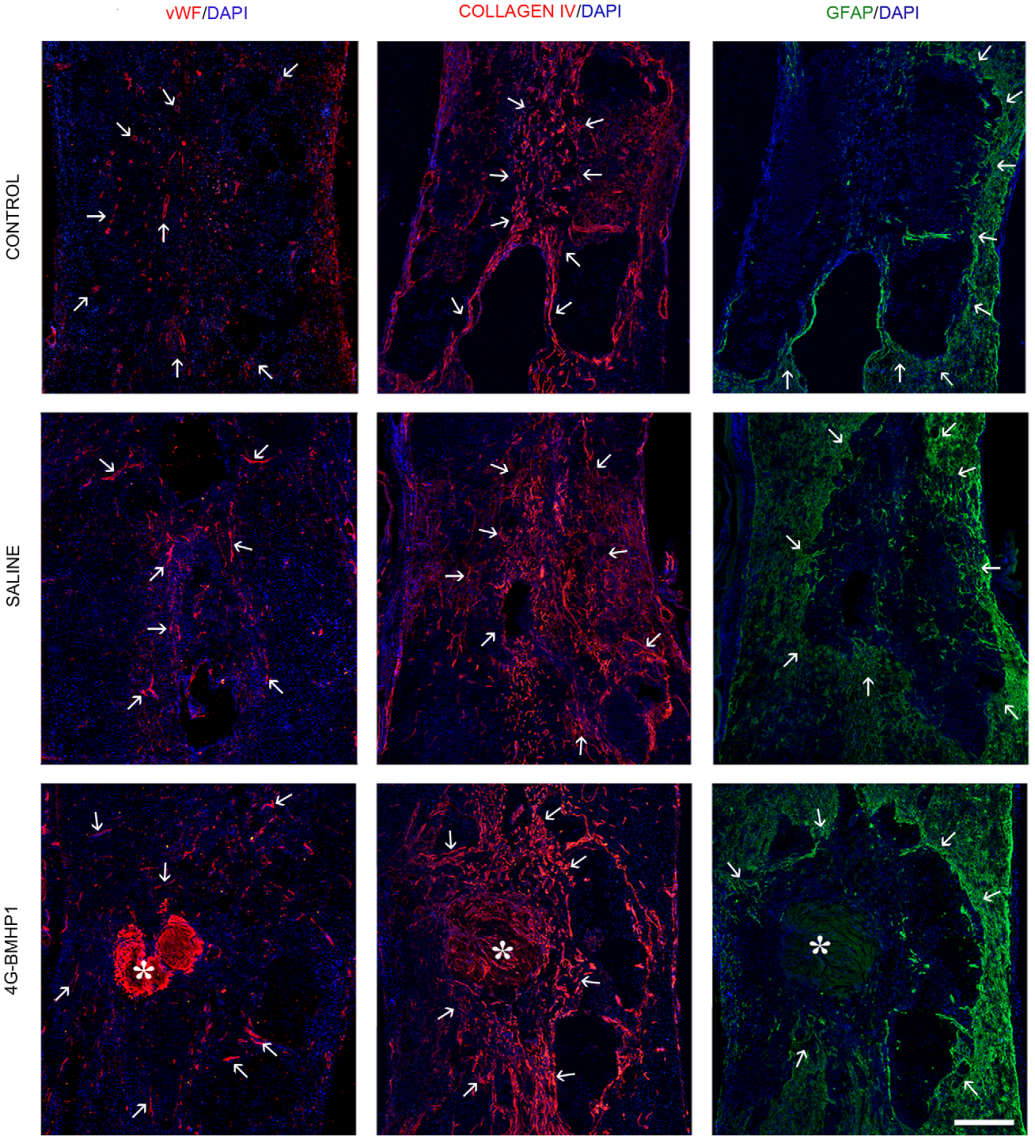
Evaluation of gliotic and angiogenic processes within the lesion area. From the left to the right, arrows indicate blood vessels (vWF+, red) infiltrating the lesion area, collagen IV deposition (red) within the cyst and the glial scar (GFAP+, green) surrounding the lesion site. Asterisks indicate the scaffold. Nuclei were counterstained with DAPI. Blood vessels infiltration appeared similar in all groups, but in the treatment group (4G-BMHP1) a greater deposition of type IV collagen occurred. Several GFAP+ astrocytes surrounding the cyst margins and infiltrating the lesion were observed in all groups but they were not detected within the scaffolds. Scale bar  = 400 µm. 2

The vascularisation was evaluated by performing immunofluorescence staining for the endothelial cell marker vWF and for two major components of basement membranes, laminin and type IV collagen. Within the lesion area, the blood vessels infiltration appeared similar in all groups, but in the 4G-BMHP1 group we found a greater deposition of laminin and type IV collagen (Figure 4).

Finally gliosis, that was evaluated by using immunofluorescence staining for GFAP, resulted similar in all groups at 8 weeks (Figure 4).

### Histological evaluation of cell infiltration and ECM deposition into the scaffold

**Figure 5:**
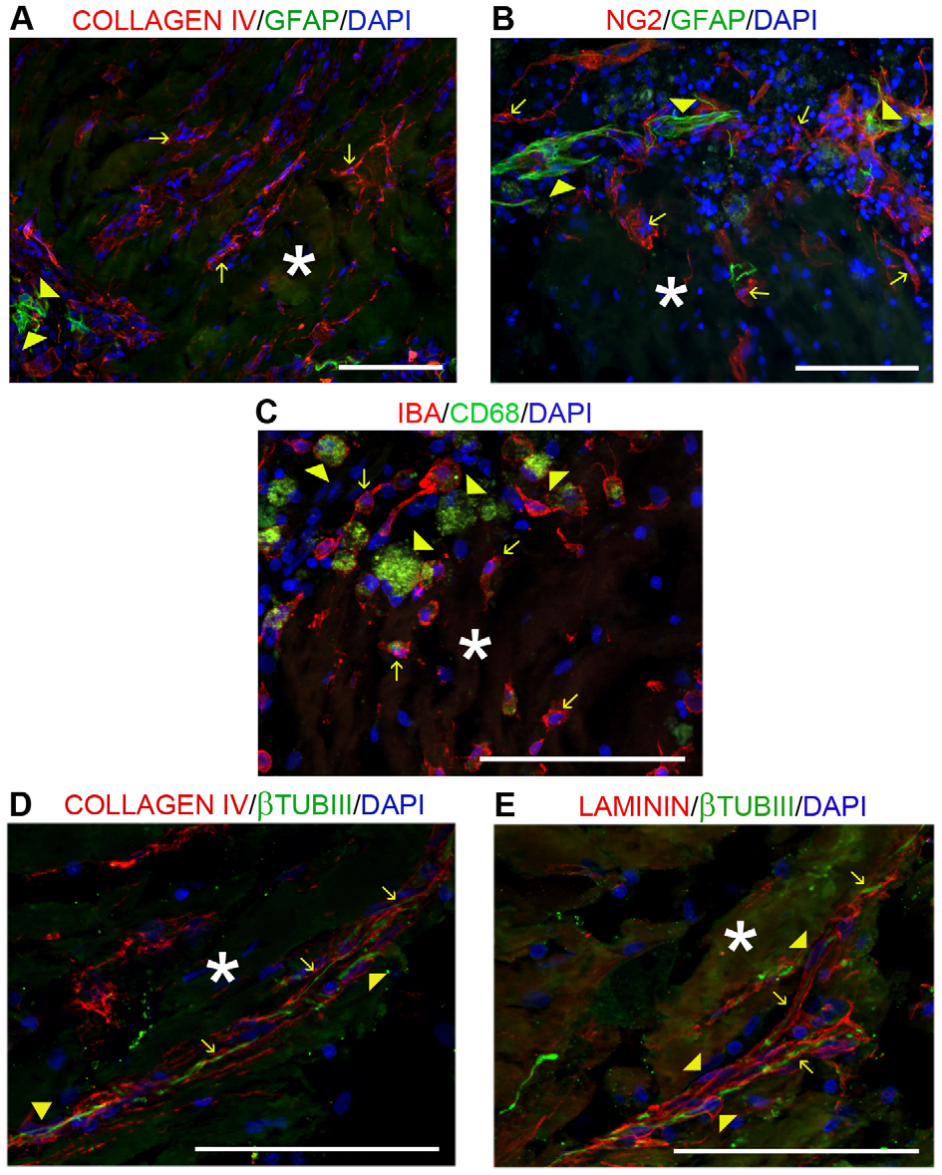
Immunofluorescence analysis of cellular and non-cellular infiltrates into the injected scaffold. (A) and (B): arrows indicate collagen IV+ cells (A, red) and NG2+ cells (B, red), whereas arrowheads indicate GFAP+ glial cells (A and B, green). (C): arrows show IBA+ microglia cells (red) and arrowheads CD68+ macrophages (green). (D) and (E): arrows indicate β-TubIII^+^ axons (green) surrounded by the basement membrane, composed by collagen IV (D, red) and laminin (E, red); arrowheads show collagen IV+ (D) and laminin+ (E) cells. Asterisks indicate the scaffold. Nuclei were counterstained with DAPI. A remarkable deposition of type IV collagen and laminin was observed into the biomaterial and specifically along axons. The non-neuronal cellular infiltrate was mainly represented by Iba-I^+^ microglia cells and NG2^+^ oligodendrocyte precursor cells, whereas GFAP^+^ astrocytes and CD68^+^ macrophages mostly surrounded the implant. Into the scaffold we also observed cells showing type IV collagen and laminin immunopositivity, strictly coupled to axons, likely ascribable to Schwann cells. Scale bar  = 100 µm.

Eight weeks after implantation, the scaffold was found in two-thirds of animals. The biomaterial had a globular appearance and resulted faintly autofluorescent (asterisks in Figures 3, 4, 5).

**Figure 6:**
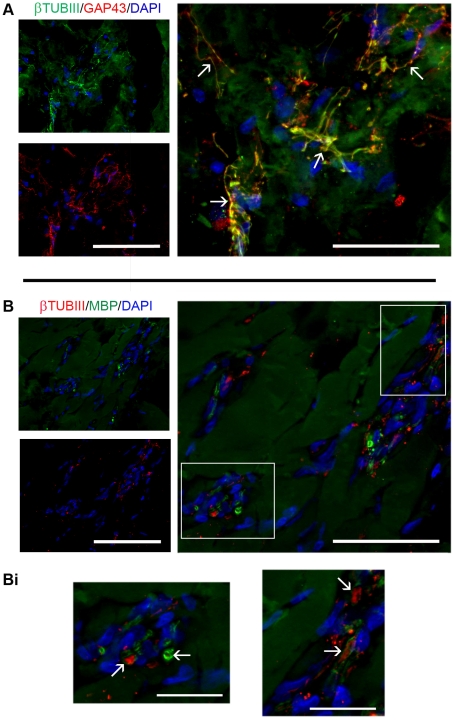
Immunofluorescence analysis of neuronal elements and nerve fibers within the scaffold. (A): the scaffold was infiltrated by neuronal cells (β-TubIII^+^, green) and sprouting/regenerating nerve fibers, that were positive for both GAP-43 (red) and β-TubIII (green). Merge of two stainings is reported at higher magnification on the right, arrows indicate some axons expressing both GAP-43 and β-TubIII. Scale bar  = 100 µm for images on the left and 50 µm for image on the right. (B): co-staining for β-TubIII (red) and MBP (green) revealed that some sprouting/regenerating fibers were myelinated, as seen on the right in the merge or below in the high-magnified inserts, where myelin surrounding some GAP-43^+^ axons is visible (Bi, arrows). Scale bar  = 100 µm for images on the left, 50 µm for image on the right and 20 µm for images below. Nuclei were counterstained with DAPI.

Within the implant we noticed several cells and nerve fibers. The majority of cells stained for Iba1, a marker for microglia, however some cells were positive for β-TubIII and for NG2, indicating the presence of neuronal elements and oligodendrocyte precursor cells, respectively; astrocytes (GFAP^+^) and macrophages (CD68^+^) were mainly found around the implant (Figure 5A, B, C). Within the scaffold we observed also laminin and type IV collagen deposition (Figure 5A), that was often associated with axons (Figure 5D and 5E, arrows). Laminin and type IV collagen immunopositivity was further associated with specific cells surrounding axons (Figure 5D and E, arrowheads), probably ascribable to Schwann cells migrated from dorsal roots [50]. Neural fibers co-stained for GAP-43 and β-TubIII (Figure 6A). Of note, occasionally these fibers were also positive for MBP, indicating that myelination occurred in some regenerating axons (Figure 6B).

### Assessment of locomotor improvements induced by the scaffold after SCI

The progressive locomotor recovery after SCI was followed by using the Basso, Beattie, Bresnahan (BBB) test [51]. BBB is an open field behavioural assessment that specifically evaluates locomotor outcome of rats after thoracic spinal cord contusion. BBB rating scale ranges from a minimum of 0 (no observable hindlimbs movements) to a maximum of 21 (plantar stepping, coordination and trunk stability like healthy rats).

**Figure 7:**
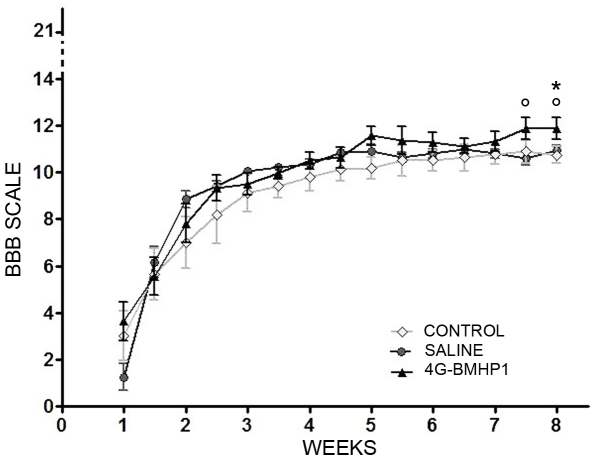
Evaluation of locomotor recovery over 8 weeks after SCI. BBB rating scale ranges from a minimum of 0 (no observable hindlimbs movements) to a maximum of 21 (presence of consistent plantar stepping, coordination and trunk stability). At 7.5 weeks after injury, in the treatment group (4G-BMHP1) we observed a better improvement of hindlimbs motor recovery compared to both control groups (saline and SCI control). This statistically relevant difference among scores implies that all groups had frequent or consistent plantar stepping but in the treatment group occurred also an occasional forelimbs-hindlimbs coordination. Values represent means ± SEM. Significance symbols: * p<0.05, 4G-BMHP1 vs SCI control; ° p<0.05, 4G-BMHP1 vs saline.

The scaffold induced, starting from 7.5 weeks after injury, a better improvement of hindlimbs motor recovery compared to both control groups (Figure 7). Saline and SCI control groups reached the maximum score (11±0.21 and 10.88±0.48 respectively) around the 7^th^ week. In the BBB test the score reached by control groups corresponds to plantar stepping, occasional or frequent, without any forelimbs-hindlimbs coordination. BBB score of saline-injected group are very similar to SCI control group, suggesting that injection into the spinal cord neither caused an important damage nor affected locomotor recovery. Rats treated with RADA16-I-4G-BMHP1 achieved the maximum score (11.86 ±0.47) around the 7^th^ week. The BBB score obtained by biomaterial-injected group corresponds to frequent or consistent plantar stepping with occasional forelimbs-hindlimbs coordination. The observed difference in locomotor coordination among treatment and both control groups was statistically relevant (P<0.05).

## Discussion

In a previous study we showed RADA16-4G-BMHP1 to be a good candidate for nervous tissue engineering as it enhanced *in vitro* NSC survival and differentiation [37]. In the current work we evaluated its neuroprotective and neuroregenerative potential when injected into the rat acute spinal cord injury.

Overall, the scaffold didn't show a relevant neuroprotective effect, as we didn't observe an attenuation of inflammatory processes nor a lesser extent of cyst and cavities in treated animals compared to untreated ones. On the other hand, the presence of the functionalized biomaterial didn't elicit an important immune response as, with the exception of IL-6 and TACE whose mRNA levels were upregulated only in the treatment group, the other inflammatory factors TNFα, iNOS and nNOS were found upregulated in both biomaterial and saline injected groups. This observation indicates that the injection procedure, even if less invasive than other implantation techniques [8], [52], can have induced a tissue loss and cell death due to needle insertion and can be then resulted in a slight increment of the sub-acute inflammatory response. The increment of IL-6 and TNFα mRNA expression can explain also the increment of GFAP and BMP2 mRNA expression seen in biomaterial and saline injected groups, because these pro-inflammatory cytokines induce the activation and migration of microglia and astrocytes [53]. TNFα also upregulates the expression of BMP2 in astrocytes [54]. However, the observed increment of mRNA expression of pro-inflammatory and pro-astrogliosis genes at 7 dpi didn't lead to relevant anatomopatological changes at 8 weeks after SCI. As matter of fact, histological analysis revealed the presence of the glial scar and of several macrophages infiltrating the lesion area in treatment and both control groups. Of note, at this time astrocytes appeared to border the cavity but not to infiltrate the scaffold. The high number of macrophages we measured at 8 weeks in all groups was similar to the value that is generally assessed in the acute phase of SCI [53], but others authors have showed in rats the presence of a second peak in the macrophage/microglial response at 60 days after moderate contusion [55]. Thus the quantity of macrophages we observed may correspond to the second peak occurring in the animal model we used.

In conjunction with the upregulation of mRNA expression of pro-inflammatory cytokines, at 7 dpi in the treatment group we observed also a higher mRNA expression of the proteolytic activators (MMP-3 and MMP-14) of MMP-9 and MMP-2 and a corresponding downregulation of two inhibitors (TIMP-1 and THBS-2) of these proteases, suggesting that the scaffold promoted the MMPs activity. MMPs are secreted by activated astrocytes, microglia and macrophages, which after a CNS damage are induced by cytokines, comprising TNFα, to produce the proforms of MMPs and their activators (other proteases or free radicals) [44]. During the acute phase of SCI, MMP-2 and MMP-9 play a critical role in the disruption of the blood-spinal cord barrier, resulting in inflammatory cells infiltration, hemorrhage and edema [56], but successively they participate in reparative processes like ischemia-induced angiogenesis and neurite outgrowth [45], [46]. These regenerative effects displayed by MMPs in the sub-acute and chronic phases of SCI well correlate with the higher mRNA levels of GAP-43 and VEGF we found at 7 dpi in the treatment group in comparison with controls. VEGF is a potent stimulator of angiogenesis and has been showed to provide a neuroprotective effect in experimental models of SCI [43]; notably, many researchers have demonstrated a correlation between VEGF release and MMP-2 and MMP-9 expression [45], [56], [57]. Thus the scaffold could have induced a bigger expression of TNFα and its activator TACE that resulted in enhanced MMPs activity and, at last, in induction of pro-angiogenic events. However, 8 weeks after SCI the histological analysis revealed a similar density of vWF positive blood vessel among groups, even if in treated animals blood vessels converged on the implant, suggesting that it could work as trophic and mechanical support for vessels. Of note, in the treatment group we observed a bigger deposition of laminin and type IV collagen, that were present also within the implants. In experimental models of SCI it has been reported that basal lamina plays a role in preventing CNS axon regeneration [58], but in human SCI it has been displayed that type IV collagen and laminin, two components of basement lamina, were mostly produced by migrating Schwann cells and fibroblasts and that basal lamina supported outgrowth of peripheral nervous system axons from lesioned nerve roots [59]. Moreover King and colleagues, after implantation of collagen, fibrin or fibronectin gels into the damaged rat spinal cord, observed that within the implants the laminin deposition often took a tubular appearance and that the majority of these laminin positive tubules were not associated with blood vessels but with axons, suggesting a role of the laminin for axonal guidance [52], [60]. By histological analysis we obtained similar results: within implants and cavities the laminin and type IV collagen were rarely found along blood vessels (marked with vWF) whereas they were often found in association with β-TubIII positive nerve fibers. Interestingly, within the scaffold we observed not only laminin deposition but also laminin positive cells surrounding axons, suggesting the presence of invading Schwann cells migrated from dorsal roots [50]. As already described by Tsiper and colleagues [61], on the surface of these laminin positive cells an accumulation of type IV collagen was also found.

Another important protein that we found upregulated at 7 dpi in biomaterial-treated animals was GAP-43. The higher GAP-43 mRNA levels compared to controls can be explained by the concurrent increased mRNA expression, in the treatment group, of MMPs activators, as these proteases can have potentially beneficial effects on axonal regeneration after SCI: MMPs favour axon regeneration by degrading various inhibitory extracellular proteins and their expression is correlated with areas of increased axonal outgrowth after CNS injury [46]. Of interest the overexpression of GAP-43 mRNA at 7 dpi resulted in an increased immunopositivity to GAP-43 at 8 weeks: in treated animals the percentage of GAP-43 positive fibers within the cyst augmented more than twofold in comparison to controls. GAP-43 is a major constituent of the growth cones and plays an important role in axonal regeneration after CNS injury [62], but it seems not to be a sufficient determinant to promote axonal outgrowth [38]. Thus to explain the supplementary axonal sprouting and/or regeneration seen in treated animals we can suppose that the scaffold could have promoted not only an overexpression of GAP-43 but also a growth permissive environment around and into the lesion site. This hypothesis is supported by the observed elevation at 7 dpi in biomaterial-injected animals of mRNA levels of CNTF, a trophic factor having neuroprotective effects in hemisection or contusion models [41]. Moreover the scaffold can have constituted a physical support for growing axons into the cyst, as suggested by the presence of several infiltrating neural fibers within the implant. Further histological analyses revealed that some of GAP-43 positive fibers found into the scaffold were myelinated. In addition we examined the phosphorylation state of neurofilament H proteins, as a marker of axonal maturity, and we found both immature (SMI-32^+^) [48] and mature (SMI-31^+^) [49] fibers.

Besides allowing nerve fibers ingrowth and basement membrane deposition, histological analysis revealed that the biomaterial promoted also migration of cellular elements, mainly composed by microglia cells, neuronal cells and, interestingly, NG2 positive cells. NG2 expressing cells are an endogenous cell population that are thought to play a role in generating new oligodendrocytes after CNS injury [63] and it has been demonstrated by Kim and colleagues that these cells proliferate and differentiate into oligodendrocytes after delivery of VEGF to the injured spinal cord tissue [43]. In the light of these studies, the myelin that we observed around few axons infiltrating the implant could have been produced, other than by migrating Schwann cells, by oligodendrocytes derived from the differentiation, probably enhanced by VEGF, of NG2 positive precursor cells.

The nervous tissue ingrowth enhanced by the scaffold resulted in a slight but statistically significant improvement of both hindlimbs motor performance and forelimbs-hindlimbs coordination in biomaterial-injected group in comparison to the spontaneous recovery observed in both control groups. As this difference appeared around one week before sacrifice of animals, it could be useful in future experiments to prolong the observation period of other weeks in order to follow the progress of this gap among groups.

Altogether, the results of the present study indicate that the injectable SAP based scaffold provided a good substrate enhancing matrix remodelling, release of trophic factors, cell migration and basement membrane deposition, resulting at 8 weeks after lesion in an increased number of regenerating/sprouting axons in comparison to the limited axon outgrowth normally occurring after incomplete SCI. Moreover the functionalized SAP showed to be compatible with the surrounding nervous tissue and to be able to at least partially fill the cavities at SCI site. Thus the functionalization of RADA16-I with a bone marrow homing motif, improved by the insertion of a longer glycine-spacer, can produce a biomimetic scaffold useful for regenerative therapy applications. Of note, as RADA16-I based scaffolds have been successfully used for NSCs culture and differentiation [33], [37] and for the sustained release of active cytokines [20], [36], RADA16-4G-BMHP1 could also constitute a good matrix for the *in vivo* delivery of growth factors and/or NSCs into the injured CNS.

## Materials and Methods

### Ethics statement

All procedures involving animals were performed according to EU Directive (86/609/EEC), to Italian legislation on animal experimentation (Decreto L. vo 116/92) and to protocols approved by the Animal Care and Use Committee of the University of Milano-Bicocca.

### SAP synthesis and purification

SAPs were prepared as previously reported [36]. Briefly, the peptide RADA16-I-4G-BMHP1 (Ac-RADARADARADARADAGGGGPFSSTKT-CONH2) was F-moc synthesized via a Liberty microwave automated synthesizer (CEM, Matthews USA). Sample masses were verified via a MALDI-TOF mass-spectrometer (Applied biosystems, Carlsbad, California), HPLC purified (Waters, Milford Massachusetts), and lyophilized (Labconco, Kansas City Missouri). Trifluoroacetic acid salts, arising from synthesis and purification of SAPs, were replaced with Cl salts via dissolution in 0.1 M HCl solution. SAPs were lyophilized and dissolved at 1% (w/v) concentration in sterile distilled water (Gibco). SAP solution was sonicated for 30 min prior to usage.

### Experimental groups

We randomly divided 45 female Sprague-Dawley rats weighing 220–250 g (Charles River Laboratories) into 3 groups as follows: 1) animals receiving only contusion (SCI control group) (N = 15); 2) animals receiving contusion and injection of saline alone (saline control group) (N = 15); 3) animals receiving contusion and injection of SAPs (4G-BMHP1 treatment group) (N = 15). For each experimental group, 8 animals were used for gene expression analysis and sacrificed 3 or 7 dpi, while 7 animals were used for histological analysis and sacrificed 8 weeks after injury.

### SCI induction and biomaterial delivery

**Figure 8:**
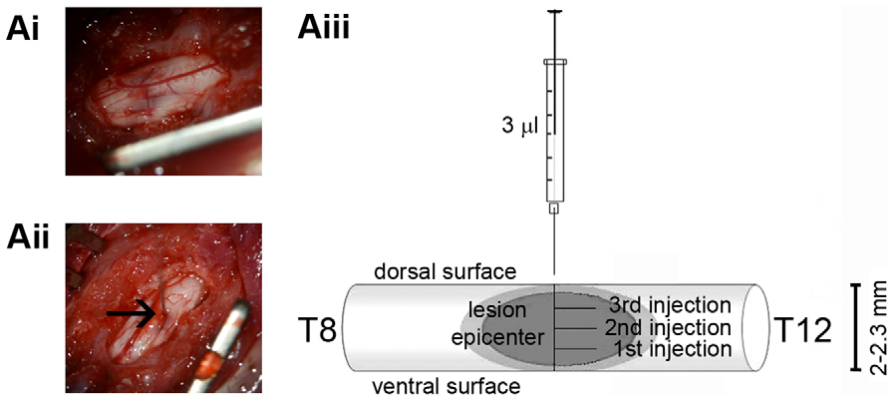
Delivery of the biomaterial into the damaged rat spinal cord. Rat spinal cord was exposed at T9-T10 level and subjected to moderate contusion using MASCIS impactor (Ai), then animals were injected with the biomaterial using an Hamilton syringe fixed at a micromanipulator (Aii, arrow indicates the needle penetrating into the injury site). The needle was inserted until reaching the ventral surface of the spinal cord then, starting from this position, the biomaterial was delivered into the lesion epicenter with multiple injections equally spaced of approximately 500 µm, for a total dose of 3 µl (Aiii).

 Rats were anesthetized with an intraperitoneal injection of ketamine (80 mg/kg) and xylazine (10 mg/kg). After laminectomy at T9-T10 level, the exposed spinal cord was lesioned by a 10-g rod dropped from height of 25 mm by using a MASCIS Impactor device (WM Keck Center for Collaborative Neuroscience, Rutgers University), as previously described [64]. Injury induced by the impactor at this height corresponds to a moderate lesion in the spinal cord [51] (Figure 8Ai). The impact velocity and compression were monitored and recorded to guarantee consistency among animals. Immediately after injury, animals were injected either with RADA16-I-4G-BMHP1 (1% aqueous solution) or saline using an Hamilton syringe (33-gauge needle) fixed at a micromanipulator: the dura mater located on the injury site was opened by performing a longitudinal cut, then either SAPs or saline was delivered into the spinal cord lesion at 3 intervals ranging about 500 µm, and at each interval two injections of 0,5 µl each were made, for a total dose of 3 µl (Figure 8Aii-Aiii). After injection, the syringe was left in the cord for 5 min, then the needle was withdrawn, the muscles were sutured and finally the skin was closed with wound clips. Rats were treated daily for one week with analgesic (carprofen, 5 mg/kg) and antibiotic (enrofloxacin, 5 mg/kg). Animals were monitored for autophagia and their bladder was manually expressed until recovery of the voiding reflex.

### Behavioral tests

Hindlimbs recovery was assessed by using the Basso, Beattie, Bresnahan (BBB) Locomotor Rating Scale [51]. Starting from 1 week post injury, every 3–4 days each rat was observed and recorded with a digital video camera for 4 min in an open field.

### Gene expression analysis

Semi-quantitative RT-PCR was employed to evaluate mRNA expression of genes of interest. For each time point studied - 3 and 7 dpi- 4 animals from each group were used.

Rats were anesthetized by an overdose of avertin (400 mg/kg) and sacrificed. A 1 cm-length of the spinal cord centered at the injury site was removed, snap-frozen in liquid nitrogen and stored at −80°C until further processing.

Total RNA was extracted using Trizol™ Reagent (Invitrogen) and treated with DNase-I, Amplification Grade (Invitrogen) according to manufacturer's protocols. Integrity of each sample was examined by agarose gel electrophoresis. Before and after DNase-I digestion, total RNA was quantified using the ***NanoDrop*** **®** ***ND-1000 spectrophotometer***. 1 µg of total RNA was used for cDNA synthesis using the SuperScript™ III Reverse Transcriptase kit (Invitrogen) according to the manufacturer's protocol.

Primers sequences were obtained from others publications [65], [66] or designed on the basis of gene and mRNA sequences available online (http://www.ncbi.nlm.nih.gov/gene). Primer design was performed using Primer3 free software (http://frodo.wi.mit.edu/primer3/). Primers were designed to be intron-spanning when possible. For each primer couple we first established the cycle number corresponding to the exponential phase of amplification of the PCR product. This evaluation was done on 50 ng of cDNA derived from intact or lesioned spinal cord tissue and PCR was performed as described below. In Table 1 we reported, for each gene of interest, forward (F) and reverse (R) primer sequence and their NCBI accession number, amplicon length and cycle number at the exponential phase.

PCR was carried out by using *Taq* DNA Polymerase, recombinant (Invitrogen) in a total volume of 25 µl containing 50 ng of cDNA and 10 µM of sense and antisense gene-specific primers. The amplification products were analyzed by electrophoresis on a 2% agarose gel in 1x TAE buffer (40 mM Tris–acetate and 1 mM EDTA) containing 0.5 µg/ml ethidium bromide. Gel images were captured using the KODAK Gel Logic 200 Imaging System.

The relative expression of genes was determined by measuring the band intensity and using cyclophilin A as housekeeping [67]. The quantification procedure based on digitalization of the PCR product after separation on agarose gel provides a well-established and sensitive method to detect even small differences in amounts of mRNA from different biological samples [68]. The densitometric analysis of bands was performed using the LabImage 1D software (Kapelan). To minimize variations among gels, each band intensity was first divided by the band intensity of the 500 bp fragment of a 100 bp DNA ladder (New England BioLabs), then the relative level of mRNA expression was calculated by dividing the band intensity of each gene by the band intensity of the housekeeping gene, as previously described [69].

### Histochemical and immunofluorescence analysis

Eight weeks after injury 5 rats from each group were deeply anesthetized with an overdose of avertin (400 mg/kg) and sacrificed by transcardial perfusion with 4% paraformaldehyde in PBS. The spinal cord segment spanning between T8 and T12 was taken, post-fixed in 4% paraformaldehyde, cryoprotected in sucrose, embedded in OCT compound and frozen. Longitudinal sections (16 µm thickness) were serially collected on a freezing microtome and stored at −20°C.

For histochemical analysis slices were processed with hematoxylin-eosin staining.

For immunofluorescence staining slices were washed with PBS, permeabilized with 0,1% Triton X-100 and blocked with 10% normal goat serum. Afterward, the following primary antibodies were applied overnight at 4°C: anti-SMI-32 (1∶1000, Covance, SMI-32R); anti-SMI-31 (1∶1000, Covance, SMI-31R); anti-βIII-Tubulin (β-TubIII) (1∶400, Covance, MMS-435P or PRB-435P); anti-myelin basic protein (MBP) (1∶350, Covance, SMI-99P); anti-growth associated protein 43 (GAP-43) (1∶200, Millipore, AB5220); anti-Von Willebrand factor (vWF) (1∶500, DakoCytomation, P0226); anti-glial fibrillary acidic protein (GFAP) (1∶500, Millipore, MAB3402); anti-NG2/ chondroitin sulfate proteoglycan (1∶200, Millipore, AB5320); anti-laminin (1∶200, Sigma-Aldrich, L9393); anti-collagen IV (1∶100, Cedarlene, CL50441AP); anti-CD68 (1∶500, Serotec, MCA341R) and anti-Iba-I (1∶1000,Wako, 019-19741). The slides were washed in PBS three times, primary antibodies were then probed with Cy3- (1∶1000, Jackson) or Alexa 488- (1∶500, Invitrogen) conjugated secondary antibodies. Sections were washed in PBS three times, counterstained with DAPI and mounted with FluorSave reagent (Calbiochem).

Brightfield and fluorescence images were acquired with Zeiss Axioplan 2 microscope or, for images at high magnification in z-stacking, with Zeiss ApoTome Microscope.

Quantification of the lesion size, macrophage infiltration and axonal sprouting/regeneration at the injury site was performed on longitudinal sections using Image J software (available: http://rsb.info.nih.gov/ij/) as described below. Sections started from the dorsal surface of the spinal cord and were spaced 160 µm.

Cavities size was quantified in seven-to-sixteen sections per animal, depending on the cyst extent. Sections were stained with hematoxylin/eosin and images were captured at 5x magnification, then single images were jointed with Photoshop in order to obtain an image of the whole cyst. The resulting image was then processed with Image J software: the cyst area was selected, the colour image was converted in binary image and the content of positive pixels was measured in order to quantify cavities size into the cyst. Also the whole cyst size was measured. Pixel area was converted to mm^2^ and measurements of either the whole cyst or cavities into the cyst of all sections were summed to produce the cumulative area of each animal.

Macrophage infiltration within the lesion area was quantified in three sections per animal. After immunofluorescence staining for CD68 and labeling of nuclei with DAPI, images were captured at 20x magnification. For each section, 16 images of the cyst area were taken for a total of 2.3 mm^2^. Cells count of CD68 positive inflammatory cells was made with Image J. The average of the measurements performed in three sections constituted the value of the number of macrophages/mm^2^ of each animal.

GAP-43^+^ sprouting/regenerating axons were quantified in six sections per animal. After immunofluorescence staining for GAP-43, images of the whole cyst area were captured at 20x magnification. Images were jointed with Photoshop and processed with Image J: the cyst area was selected, the colour image was converted in binary image and the content of positive pixels was measured in order to quantify the GAP-43 positive area into the cyst. The mean of the six measurements, expressed as percentage of the total area of the cyst, constituted the value of the axonal regeneration of each animal.

### Statistical analysis

Data were processed using GraphPadPrism 5 software. Values were reported as means ± standard error of the mean (SEM). For BBB scores and gene expression analysis, multiple and pairwise comparisons among groups were performed by one-way ANOVA and Tukey test.

For quantification of the cavity size, macrophage infiltration and axonal regeneration, comparison between the treated group and each control group was made by Mann-Whitney test. All analyses were two-tailed and p values <0.05 were considered as statistically significant.
